# Occult Metastasis: Incidence, Pattern, and Impact on Survival in Patients with Oral Cancer, pN0 vs pN1 in a Cohort of cN0. A Prospective Cohort Study

**DOI:** 10.1007/s12070-024-04968-2

**Published:** 2024-08-19

**Authors:** Kishore Das, Gopi Satya Sai Reddy Gontu, Kanato Aasumi, Raj jyoti Das, Anupam Das, Tashnin Rahman, Ashok Kumar Das, Kaberi Kakati

**Affiliations:** Department of Head & Neck Surgery, Dr B Borooah Cancer Institute, Flat No: B5, Guwahati, Assam, 781016 India

**Keywords:** Oral cancer, Neck dissection, Curves, Kaplan Meier survival

## Abstract

Squamous cell carcinoma of the oral cavity is the most common malignancy of head and neck cancer in India. With nodal dissemination, there is a significant decrease in survival. This study is aimed at studying the impact of occult metastasis on an estimated overall survival in clinically N0 patients post neck dissection. In a prospective study with 110 patients with squamous cell carcinoma of the oral cavity, clinically N0 with all T stages were included in the study. operated on for both primary and neck followed up for a median of 5 years after completion of definitive treatment. The impact of surgery on patients with occult metastasis was questionable in our study with a difference in estimated overall survival of around 11 months, which was not significant statistically. Subset analysis had shown the correlation of overall survival with a grade of tumor, T stage and depth of invasion which was statistically insignificant. Correlation of occult metastasis with the depth of invasion, T stage was statistically significant. we conclude that even after addressing the neck in clinically N0 patients with oral cancer, there is a difference in the overall survival of the patients with occult metastasis which might not be statistically significant with a p-value of 0.68. We conclude that with neck dissection in patients with occult metastasis, improved survival might not be comparable to pathological N0.

Level of Evidence: LEVEL -2.

## Introduction

Cancer is a prominent cause of death and a significant impediment to extending life expectancy in every country. According to WHO estimates, cancer is the primary or second major cause of death before the age of 70 years in 112 of 183 countries and third or fourth in another 23 [1]. Squamous cell carcinoma is the most common oral cancer, with an estimated global incidence of 275,000 new cases each year, with an increase in South East Asia [2]. In India, carcinoma lip and oral cavity is the second most common site of cancer with a 10.3% share in total cancers following breast cancer, with male predominance (16.2% vs 4.6%) [1, 3]. Tobacco and alcohol consumption remain the most dominant etiologic factors in oral cancer in India [4].

Surgery is the treatment of choice for oral cancer, and the cervical lymph node status is the most important prognostic factor in the treatment of patients with oral squamous cell carcinoma, as even a single lymph node metastasis can reduce survival and significantly alter the postoperative course of treatment [5]. Therefore, meticulous clearance of cervical lymph nodes plays a crucial part in the treatment of oral cancer. It was George Crile who systematically described the technique of radical neck dissection in 1906 [6]. Suarez was the first to describe a systematic approach to modified radical neck dissection and Bocca popularized modified radical neck dissection [7, 8]. Then came a lot of debates about when and where to address the neck including the N0 neck in early-stage oral cancers which were settled by the N0 neck trial which showed the results in favor of elective neck dissection [9]. SENDS trial, a replica of the N0 neck trial did draw the same conclusion of preferring elective neck dissection over observation in early oral cancers [10]. Occult metastasis does effect the survival but do have better prognosis than clinically positive neck node [11]. The prognosis of the patients in terms of overall survival lies in between pathological N0 and pathological N1. Elective neck dissection in case of occult metastasis does improve the prognosis but if it is comparable to pathological N0 or not is yet be elucidated [9, 10].

In this study, we did try to fill the gap in the literature regarding the improvement in overall survival post neck dissection in clinically N0 patients and if improved how much will it be in pathological N0 in comparison to pathologically N1.

## Aims

To study occult metastasis and its impact on oral cavity cancers.

## Objectives

1. To study the incidence of occult metastasis.
2. To study the pattern of occult metastasis concerning the levels of lymph nodes, DOI (Depth of Invasion) and grade of the tumor.
3. To study the impact of occult metastasis on survival post elective neck dissection.

## Materials

As per strobe guide lines.

### Study design

Prospective cohort study.

Setting: DR. BHUBANESHWAR BOROOAH CANCER INSTITUTE (BBCI).

Locations: GUWAHATI.

Periods of recruitment: 2018–2021.

Median follow-up: 5 years.

Data collection: on every follow-up, usually at 3 months.

## Inclusion Criteria

Age: 18–80 years.

Sex: any.

Diagnosis: histologically proven oral squamous cell carcinoma (all 8 subsites, any grade).

Stage: c T (1–4) N0M0 who are amenable to definitive treatment.

Laterality: Right, Left, Both.

Previous treatment for head and neck cancer: nil.

Previous diagnosis of head and neck cancer: nil.

Second primary: NIL.

## Exclusion Criteria

### AGE

Less than 18 and more than 80.

Cases who received any previous treatment for head and neck cancer.

re-operated, Residual/recurrent cases/the second primary.

Those cases where the neck has been operated on before for other diseases,

Previous treatment history for head and neck cancer with in 5 Years.

Second primary: Present.

Vulnerable groups: pregnant females, mentally challenged.

### Sample Size

A total of 110 patients who gave valid consent and who fulfilled the inclusion and exclusion criteria were taken up for the study.

### Variables Defined in the Study

Diagnostic criteria: Histopathological report from the biopsy with WDSCC/MDSCC/PDSCC.

Occult metastasis: clinically and radiologically node-negative with post-operative final histopathology report showing nodal disease were considered as positive for occult metastasis.

Recurrence: histopathological (biopsy proven) lesion after 3 months of disease-free interval after definitive treatment.

Grades: Well differentiated squamous cell carcinoma (WDSCC).

Moderately differentiated squamous cell carcinoma (MDSCC).

Poorly differentiated squamous cell carcinoma (PDSCC).

### Sites Of Oral Cavity Included For The Study

All 8 subsites as per AJCC 8.

### Staging

As per the latest AJCC8/ CAP PROTOCOLS.

### Levels of Neck

As per AJCC 8.

### Depth of Invasion

Pathological from the final histopathology report, defined as the deepest level of invasion from the reconstructed mucosal surface.

## Methods

Patients who are eligible for the study are included in the study after taking informed consent. Patients were operated on as per the current standard guidelines of wide local excision for the primary, modified radical neck dissection for the neck, and local/ regional/ free flap reconstruction as decided by the JOINT TUMOR BOARD. Following the final histopathology report, patients were treated with radiation/ concurrent chemoradiation/ observation as decided by the JOINT TUMOR BOARD.

Investigations to rule out second primary: Upper GI Endoscopy.

Investigations to rule out distant metastasis: HRCT Thorax, USG abdomen.

Follow-up: patients were followed up as per the guidelines, data was recorded every 3 months during the follow-up.

Methods of follow-up: Physical till the advent of COVID-19.

Via teleconsultation during COVID-19.

During and after the 2nd phase as per the convenience of the patient either via tele-consultation or physical outpatient department (OPD).

### Tele Consultation

At BBCI, teleconsultation was done as per the institute guidelines. It is usually done via video conference call after the patient generates a request for the call either via the web portal or via telephone.

### Data Sources

- Final Histo-pathology report
- EMR/ HIS (Hospital Information System)
- OPD follow-ups
- Teleconsultation notes

### Bias/ Confounding Factors

During the times of Covid-19, In tele consultation follow ups physical examination could not be done.

### How the Bias was Addressed

We did try our best to get the patient to a nearby oncology Centre at their home, which sometimes could not be done because of logistical issues.

Grouping of quantitative variables for the study:

Grade: WDSCC, MDSCC, PDSCC.

Depth Of Invasion: 0–5MM, 5–10MM, 10–20MM, > 20MM.

Occult Metastasis: Yes, No.

Recurrence: Yes, No.

Death: Yes, No.

## Statistical Methods

1. Correlation via Pearson.
2. Chi square analysis and likely hood ratios.
3. Survival via Kaplan Meier analysis
4. Sub group analysis

Loss to follow up: We did have 4 patients who were lost to follow up and were tried to contact on the phone.

How the missing data was addressed: Till the date of follow-up patients had no recurrence.

Data missing was ignored in statistics.

Median follow-up: 29.44 months.

## Primary Outcomes

1. Occult metastasis incidence, pattern.
2. Overall survival estimated via Kaplan Meier Analysis.
3. Impact of occult metastasis on survival after addressing neck.

**Fig. 1:**
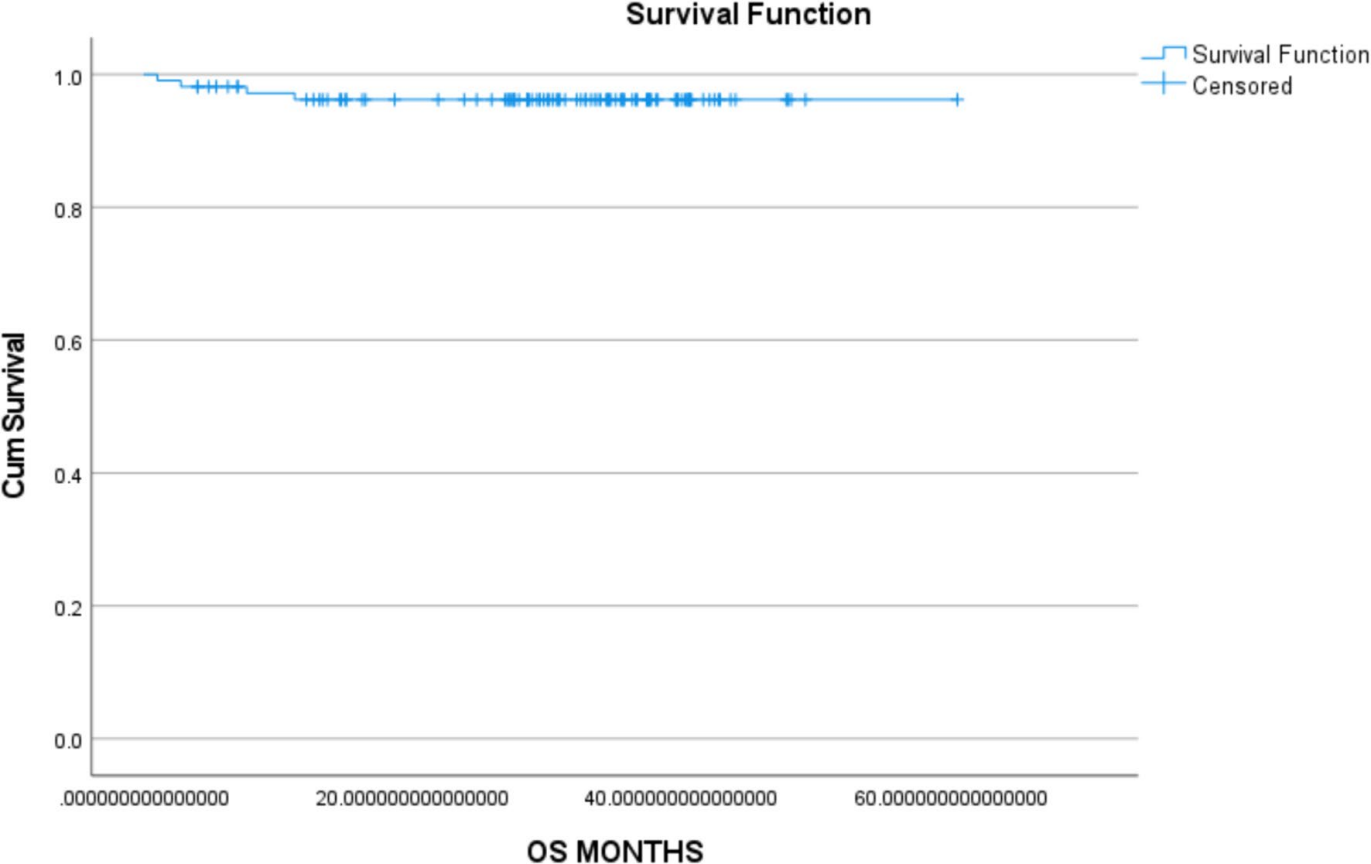
Overall survival

**Fig. 2:**
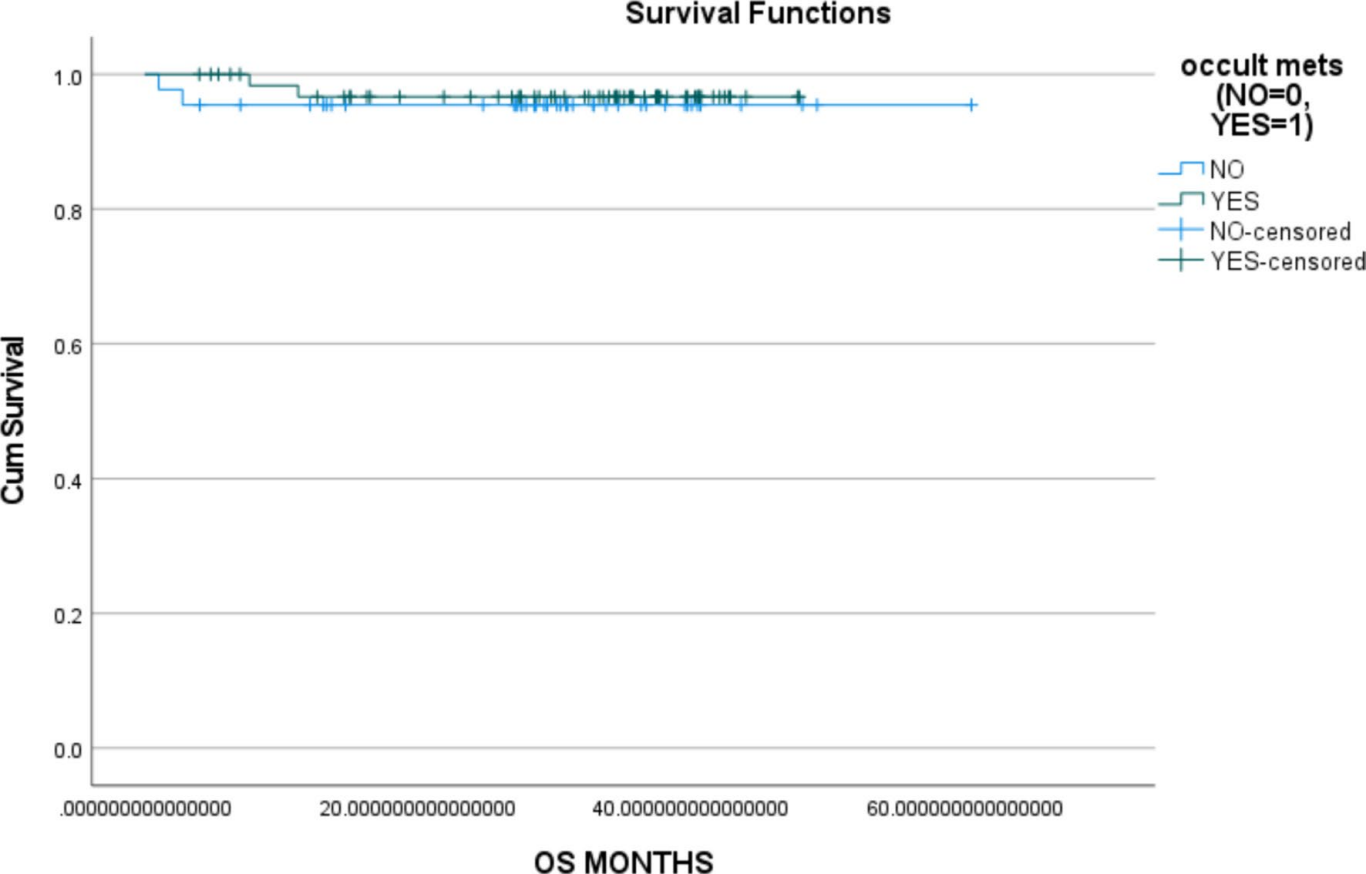
Overall survival with and without occult Mets

Descriptive statistical analysis was used to represent the data. Data were analyzed using IBM SPSS software version 21.0 with the guidance of a medical statistician. Data were summarized in the form of proportions, frequency tables, and bars for categorical variables (Figs. 1 and 2).

## Results

110 patients with Squamous cell carcinoma of the oral cavity, who came to Dr. B Borooah Cancer institute were included and analyzed in this study. The age of the patients was ranging from 31 to 75 years with a mean age of 54.1 years. Out of 110 patients, there were 61 males comprising 55.5% and 49 females comprising 44.5% of the patients (Table 1).

**Table 1:** Demographics of the study population

S.NO	Characteristic	Mean/ Total	(Min–Max)
1	Age (Mean ± SD)	54.10 ± 9.49 years	31–75 years
2	Depth of invasion (Mean ± SD)	9.09 ± 5.10 mm	2–25 mm
3	Male	61	
4	Female	49	
5	cT1	38	
6	cT2	34	
7	cT3	27	
8	cT4	11	

Median follow-up: 2.5 years (7 months–50.1 months)

Demographics of Study Population:

Incidence of Occult Metastasis: The site wise incidence and incidence in occult metastasis were as described in Table 2. We did not have any floor of mouth cancers which can be a negating point in the study.

**Table 2:** Incidence of occult Metastasis

S.no	Site	Rate	occult metastasis rate
1	Buccal Mucosa	61 (55.5%)	30.09%
2	Lower Alveolus	25 (22.7%)	16.4%
3	Oral Tongue	13 (11.8%)	6.4%
4	Upper Alveolus	5 (4.5%)	4.5%
5	RMT	3 (2.7%)	1.8%
6	Hard Palate	1 (0.9%)	1.1%
7	Lip	2 (1.8%)	0
8	Floor of mouth	0	0
	Total	110	

## Correlations

We did analyze the Pearsons co-relation of depth of invasion, occult metastasis and levels of occult metastasis which is summarized in the table below (Table 3).

**Table 3:** Correlations of occult metastasis

		Pearsons correlation/LHR	*p*-value	95% CI (2- tailed)
Depth	Occult metastasis	0.40(PC)	< 0.01	0.236–0.551
Occult metastasis	T stage	3.01 (LHR)	0.04	–
Levels of occult metastasis	depth	18.156(LHR)	0.03	–

### Overall survival

Overall survival of the whole cohort of 110 was analyzed with Kaplan Meier analysis and is given below (Table 4).

**Table 4:** Cross tabulation with occult metastasis

Variable	Chi-square value	*p*-value
Grade	0.724	0.69
T stage	5.049	0.16
DOI	4.469	0.34
Level of occult mets	6.73	0.15
Age	2.01	0.73

### Effect of occult metastasis on overall survival

#### Means and Median for survival time

Compared to the median estimate OS, the effect of occult metastasis did decrease the OS in our study but with a *p*-value of 0.68, it was statistically insignificant.

Subset analysis: Age, Grade, T stage, DOI with Overall Survival.

Comparison of stage wise disease with and without occult metastasis OS (Table 5).

**Table 5:** Stage-wise analysis

variable	OS (pN0 vs PN1)	*p*- value
T1	58 versus 56	0.43
T2	54 versus 54	0.52
T3	50 versus 47	0.64
T4	51 versus 50	0.68

## Discussion

Neck disease is an important factor in the survival of patients with oral squamous carcinoma. Neck disease Itself alone decreases survival by 50% [12]. So, prediction of lymph-node metastasis and addressing the neck rather became a need than to observe for them, to take a call for elective neck dissection. From earlier studies that did take into consideration of only tumor size and grade to the latest studies with biomarkers as predictors, many studies were done to predict the probability and pattern of lymph node metastasis in oral cancer [13, 14]. The most important predictors that have been found to have a significant relationship with lymph node metastasis are Tumor size, grade including differentiation of tumor, and depth of invasion. The new AJCC staging system (8th edition) incorporates a depth of invasion to stage oral cancers. It is a recognized predictor for neck nodal metastasis and local recurrence, but the associated risk is not well defined [15]. The concept of occult metastasis and skip metastasis became a topic of debate and were being explored a lot in recent times. In our study, we did try to find the impact of occult metastasis on the overall survival after addressing the neck, side by side any co-relation and association between the pattern of the occult lymph node metastasis with the depth of invasion, tumor grade, T stage.

The median age in our study was 54.1 years which is less than the average age of incidence based on 29 cancer-based registries in India which are about 64 years [3]. This decrease in age can be attributed to increased intake of smokeless tobacco in the northeastern part of India from a young age(20–22), in which around 66.6%cases of head and neck are usually diagnosed at an advanced stage in India [16]. In our cohort of 110 patients, 61 were Male (55.5%) and 49 females (44.5%) which does go hand in hand with GLOBOCON data 2020 of India [1]. The incidence of oral cancer for males was 64.8% and for females, it was 37.2% at 70 years of age. The next highest magnitude was observed in the west and northeast regions (58.4%) at 60 years of age [1].

Tumor location in our cohort is highest in Buccal Mucosa 61 (55.5%), followed by Lower Alveolus 25 (22.7%) and Oral Tongue 13 (11.8%). The incidence is higher in buccal mucosa and lower alveolus because most of our patients had the habit of placing tobacco in the lower gingival buccal sulcus [17]. Most of the studies on occult metastasis did take into consideration only tongue and early T stage including T1 and T2 [18]. we included all T stages without clinical and radiological nodes per se including all stages with occult metastasis to see the impact of occult metastasis on the survival irrespective of tumor stage. However, in subset analysis, we did analyze the impact of occult metastasis on recurrence and survival for both early and advanced oral cancers separately which we did find not significant statistically with a ‘p-value’ of 0.52. There’s literature quoting the worsening of survival with need for chemotherapy post-surgery in patients with occult metastasis [19]. We did find minimal literature quoting the improvement in survival post-surgery in N0 patients, which can be extrapolated from the N0 trial and SENDS trial [9, 10]. The improvement in the survival post neck dissection in patients with pathologically positive node whether it is comparable or not to the pathological N0 or is what we did try to elucidate in the current study.

The correlation between occult metastasis and T stage was found to be statistically significant with a p-value of 0.04 and LHR of 3.01, Most of the studies in literature does talk about early cancers where the correlation between tumor stage and occult metastasis was found to be consistent with our study [18]. Most of the current literature does talk about occult metastasis in tongue cancers only, In this study, we did include all 8 subsites of the oral cavity which were defined as per AJCC8 [15, 19]. In a subset analysis, we did find an insignificant relationship statistically to occult metastasis with tumor site with an LHR of 0.04 and *p*-value of 0.06.

Coming to the estimated overall survival, the primary objective of the study was to elucidate the impact of occult metastasis post neck dissection on overall survival. we did find a difference in median estimated survival of around 11 months which was found to be statistically insignificant with a p-value of 0.68. In our subset analysis, we did find no statistically significant relationship between overall survival and effect grade of tumor, stage of the tumor, depth of invasion, level of occult metastasis, and age. These findings of our study do not correlate with the available literature even though mostly on the tongue and early cancers they suggest a statistically significant improvement in the survival with neck dissection compared to the observation [9, 10]. There is minimal evidence comparing the survival of the pathological N0 to pathological N1 of clinically N0 patients post neck dissection. Even though our study has statistically insignificant results it raises a future research question in this area. This study doesn’t oppose neck dissection in early cancers as given by N0 neck trial and SENDS trial, it did try to estimate the impact of occult metastasis on overall survival in clinically N0 patients post elective neck dissection. It does give an insight in to hypothesis of evaluating the improvement in survival post neck dissection in patients with occult metastasis and comparison with clinical and pathological N1, clinical and pathological N0.

## Conclusion

We conclude that in our study to find the impact of occult metastasis on overall survival in clinically node-negative patients of oral cancer, we did find a median difference of around 11 months between pN0 and PN1 which was found to be statistically insignificant. In a subset analysis, we did find a statistically significant relationship only with the incidence and pattern of nodal metastasis which per se were the other primary objectives of the study.
